# Illustrated Magazine of Art

**Published:** 1853-01

**Authors:** 


### ILLUSTRATED MAGAZINE OF ART.

The first number of this work is just from the press, and is received only in time to refer to its general appearance. In this it fully meets our expectations, and indeed is much richer in illustrations and well written articles than we expected to see. A glance at the table of contents will give an idea of the work. First, we have a history and description of the English House of Commons. This, with the engraving of the interior of the House of Commons is worth the cost of the number. We then have a likeness of Geoffrey Chaucer, with a fair criticism of his poetic talent, justly styling him the "poet of reality." The Egyptian Fellah's, the Bird of Paradise, and Brother Alphus, a Swedish legend, give us an idea of the contents. The closing article is a magnificent illustration of the funeral procession of the Duke of Wellington.

We feel assured the Illustrated Magazine of Art, will have a very large circulation. It is issued the first of every month at $3 per year in advance, postage free. The present number contains 60 pages double columns ; and in typographical execution will compare well with any of our literary periodicals.

It is published by Alexander Montgomery, 17 Spruce st., N. Y.

